# Thrombosis and bleeding outcomes in the treatment of cerebral venous thrombosis in cancer

**DOI:** 10.1186/s12959-021-00292-9

**Published:** 2021-06-01

**Authors:** Nadia I Abelhad, Wei Qiao, Naveen Garg, Cristhiam M. Rojas-Hernandez

**Affiliations:** 1 Department of Medicine, University of Texas Health Science Center at Houston, Houston, USA; 2 Department of Biostatistics, University of Texas M.D. Anderson Cancer Center, Houston, USA; 3 Department of Diagnostic Radiology, Division of Diagnostic Imaging, University of Texas M.D. Anderson Cancer Center, Houston, USA; 4 Section of Benign Hematology, Department of Medicine, University of Texas M.D. Anderson Cancer Center, 1515 Holcombe Blvd. Suite 1464, TX Houston, USA

**Keywords:** Cerebral, Thrombosis, Cancer, Anticoagulation

## Abstract

**Background:**

There is a need for clinical outcome data of cerebral venous thrombosis (CVT) in cancer patients. We examined the recanalization, thrombosis recurrence and major bleeding during CVT treatment in a cancer exclusive adult population.

**Methods:**

We performed a retrospective review of cancer associated CVT identified through an institutional data warehouse. The primary endpoint was radiological and comprised the evaluation of thrombus recanalization at 12 months. Secondary endpoints were clinical and included rates of bleeding complications and recurrence of CVT. Variables were compared across subgroups of study outcomes. The backward stepdown procedure was used to identify variables for the final logistic model regarding thrombosis and bleeding outcomes.

**Results:**

The population included forty-five patients, slightly predominant of male adults (55.6%) with a median age of 54.5 years. Solid malignancies comprised 64.4% of cases. A total of 31 cases were treated with anticoagulation. CVT recanalization was documented in almost 60% of cases. The cerebral venous thrombosis recurrence or propagation rate at 12 months was 15.6%. Major bleeding complications were observed in 15 patients.

**Conclusions:**

Our findings are suggestive of a narrow therapeutic index of anticoagulation in cancer-CVT. Careful monitoring of anticoagulation effect and bleeding complications are of utmost clinical relevance in cancer patients. Further larger and controlled studies are needed to confirm our observations.

## Introduction

Cerebral venous thrombosis (CVT) affects approximately five people per million annually and accounts for 0.5–1 % of all cerebral thrombotic events [1]. Previously described risk factors include female gender, age < 40, pregnancy, hormonal contraceptive use and thrombophilias [2]. It has been speculated that CVT could be more frequent in cancer patients due to a variety of mechanisms including direct tumor compression, cerebral sinus invasion, chemotherapeutic or hormonal agents [3, 4].

The exact incidence of CVT in cancer only population is unknown. Up to 30 % of all first venous thromboembolic events are cancer associated and 7 % of patients with CVT have a history of cancer at time of CVT diagnoses [5, 6]. Studies assessing the association between CVT and recanalization rate, major bleeding or venous thrombosis reoccurrence in cancer only population have not been performed.

Our goal was to examine the clinical outcomes in cancer associated CVT, those included thrombus recanalization, cerebral venous thrombosis recurrence/propagation and bleeding rates.

## Methods

We examined a retrospective cohort study of cancer patients with CVT identified through an institutional data warehouse at the University of Texas MD Anderson Cancer Center Hospital between January 2002 and June 2017.

**Fig. 1:**
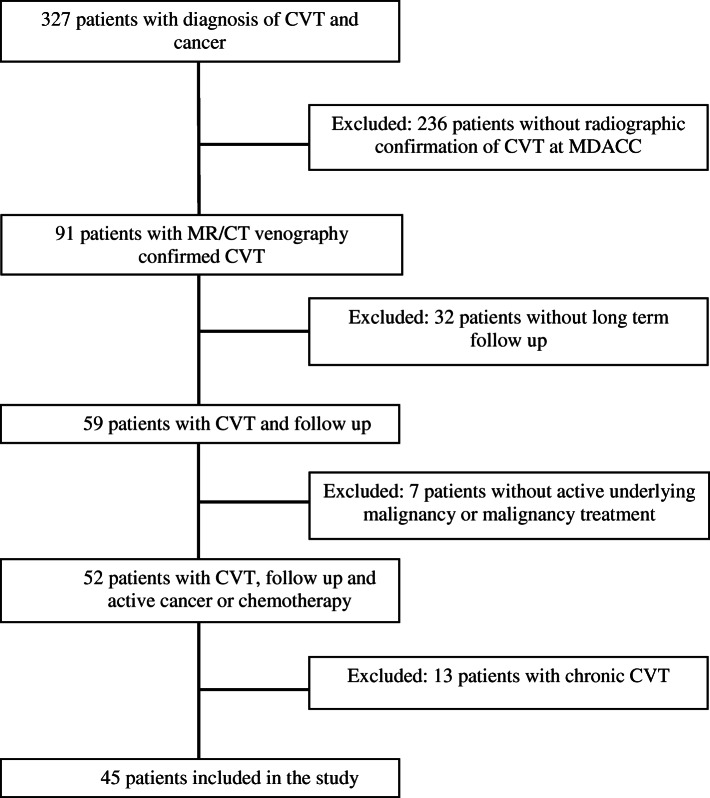
Selection  of cancer patients with acute cerebral venous thrombosis (CVT)

Adult (≥ 18 years) patients with active cancer or active cancer treatments and diagnosis of acute CVT were included. Patients with other indications for long term anticoagulation, chronic CVT, patients with acute CVT diagnosed at outside facility and not confirmed at our facility or those without documentation of clinical follow up 12 months after initial CVT event were excluded. (Fig. 1).

Diagnosis of acute CVT was confirmed in all patients with computed tomographic-venography or magnetic resonance imaging with venography. This study was approved by the local ethics committee and institutional review board.

The primary endpoint was thrombus recanalization at 12 months, defined as partial (less than complete) or complete through the review and comparison of the diagnostic imaging data by an independent neuroradiologist.

Secondary endpoints were bleeding complications and CVT recurrence/propagation at 12 months. The outcome of bleeding was obtained from medical record review and defined by the World Health Organization bleeding scale [7].

Demographic data, major surgery, trauma, hormonal contraceptive use, cancer diagnosis and therapy were obtained at the time of CVT. CVT location and associated intracranial infarction or hemorrhage at time of initial diagnosis was recorded using imaging reports. Therapeutic medical antithrombotic treatment was recorded.

The distribution of each categorical variable was summarized in terms of its frequencies and percentages. Variables were compared across subgroups of study outcomes. The backward stepdown procedure was used to identify variables for the final logistic model regarding thrombosis and bleeding outcomes. Computations were carried out in SAS version 9.4.

## Results

The population included 45 subjects, predominantly middle-aged adults and approximately half of them were overweight. Most of the patients had solid malignancies with locally advanced or distant metastatic disease (Table 1).

**Table 1:** Baseline Demographic and Clinical and Radiological Characteristics at Cerebral Venous Thrombosis (CVT) presentation

Characteristic		N (%)
Male		25(55.6 %)
Age (years) Median, [IQR]		54.5, [41.5,62.4]
Body Mass Index (Kg/m^2^) Median, [IQR]		27.2, [23.4, 33.4]
Malignancy type		
Hematologic		15 (33.3 %)
Solid		24 (53.3 %)
Primary CNS		5 (11.1 %)
Concurrent solid and CNS		1 (2.2 %)
Malignancy status		
Active		32 (71.1 %)
Remission < 2 years		8 (17.8 %)
Remission > 2 years		4 (8.9 %)
Staging (AJCC)		
1		8 (17.8 %)
2		4 (8.9 %)
3		5 (11.1 %)
4		17 (37.8 %)
Active chemotherapy		20 (44.4 %)
Hormonal cancer therapy		4 (8.9 %)
Tyrosine kinase inhibitor		1 (2.2 %)
Use of ESA		1 (2.2 %)
Recent major surgery		27 (60.0 %)
Multifocal CVT		26 (57.8 %)
Occlusive CVT		14 (31.1 %)
Concurrent brain venous infarction		12 (26.7 %)
Concurrent brain parenchyma hemorrhage		16 (35.6 %)

More than half of the cases had multifocal CVT and over one-third had concurrent intracranial hemorrhage at presentation (Table 1).

Anticoagulation was prescribed in 73.3 % of cases, with a median duration of treatment of 7.9 months. Low molecular weight heparins (LMWH) were used in 23 cases, while 10 cases were treated with warfarin.

For the evaluation of the primary outcome, repeat vascular brain imaging for comparison was available in 41 patients. In those evaluable cases, partial thrombi recanalization was achieved in 36.6 % of them, 95 % CI [23.3, 51.8]; and complete recanalization in 22.0 %, 95 % CI [11.5, 36.2].

Although recanalization was more frequently seen in patients that received anticoagulation (64.5 % versus 40.0 %), there was not a statistical significant association between recanalization and the use of anticoagulation therapy (*p* = .348).

Overall bleeding occurred in 46.7 % of patients, 95 % CI [32.7, 61.1]. A total of 15 patients suffered major bleeding defined as WHO grade 3 or 4 (Table 2).

**Table 2:** Bleeding Complications after Diagnosis of Cerebral Venous Thrombosis

Bleeding characteristics	N	%, 95 CI^a^
WHO grading of bleeding		
< 3	6	28.5, [8.4, 66.8]
3	1	4.8, [0.5, 20.2]
4	14	66.7, [45.4, 83.7]
Bleeding location^b^		
Brain	13	61.9, [40.7, 80.1]
Skin	4	19.0, [6.8, 39.2]
Body cavity	2	9.5, [2.0, 27.2]
Nasal/oral mucosa	2	9.5, [2.0, 27.2]
Gastrointestinal tract	1	4.8, [0.5, 20.2]

^a^Estimates calculated over 21 total cases of bleeding

^b^One patient presented with bleeding in the brain and other body location

Intracranial hemorrhage (ICH) was the most common major bleeding complication. Of those patients, seven presented at diagnosis of CVT and the bleeding worsened (1 with subdural hematoma, 1 case with a subdural hygroma with new blood products, 2 cases of hemorrhagic tumor in the brain and 3 with other parenchymal bleeding). The remaining six cases had ICH de novo after CVT diagnosis (1 case ICH after fall, 1 case of brain tumor hemorrhage, 1 case in the setting of acute leukemia and 3 cases after central nervous system procedures).

The occurrence of ICH during treatment for CVT did not correlate with the presence of multifocal CVT (*p* = .507), venous infarction at diagnosis (*p* = 1.000) or CVT recurrence (*p* = 1.000).

The incidence of ICH after CVT diagnosis was higher in the anticoagulation group (30.3 %, 95 CI [16.8, 47.1]) versus no anticoagulation (25.0 %, 95 CI [7.6, 52.9]) with no significant statistical difference (*p* = 1.000).

When examining the bleeding outcomes by the treatment and patient-clinical characteristics, the choice anticoagulation agent was associated with those events. For LMWH, (8/23) 34.8 % had any bleeding complications; for warfarin, (8/10) 80.0 %, (OR 7.50, 95 CI 1.05–54.3, *p* = .048).

The overall rate of CVT recurrence was 15.6 %, 95 % CI [7.2, 28.1]. The specific recurrent rates of not anticoagulated, LMWH- and warfarin-treated patients were (3/12) 25.0 %, (3/23) 13.0 %, and (1/10) 10.0 %, respectively. Those differences were not statistically significant (*p* = .362).

Since the total number of cases of CVT recurrence was small, further analyses for differences by clinical and treatment features were not performed.

## Discussion

Current guidelines recommend initial CVT management with adjusted-dose unfractioned heparin (UFH) or weight-based dose of LMWH followed by vitamin K antagonists (VKA) [8–12]. However, those guidelines, and the clinical studies supporting them, lack of a sufficient number of cases with concurrent active malignancy to fully extrapolate those recommendations to cancer population.

Recently published results from a randomized clinical trial confirmed the efficacy and safety of long-term anticoagulation for CVT with oral anticoagulants (VKA or dabigatran) [13]. Evidence-based data to support the use of other oral anticoagulants is at this point less robust [14].

Our findings suggest that the risk of thrombotic and hemorrhagic complications are both high in cancer patients who suffer CVT. Those complications are well known to be prevalent in cancer population, so there is a narrow therapeutic index when anticoagulation treatments are instituted [15].

Studies in non-cancer CVT population have shown an association between the lack of venous recanalization and worsened clinical outcomes [16]. Sousa et al. showed that recanalization was associated with a 3.3-fold increase in the odds to complete functional recovery (95 % CI, 1.2–8.9) [17, 18]. In a meta-analysis of 818 non-cancer CVT cases, the recanalization rate was 85 % (95 % CI, 80–89; I^2^ = 58 %) in patients receiving anticoagulation [16]. Proposed mechanisms for those findings include prevention of propagation of CVT, restoration of anterograde drainage and salvage of brain tissue from permanent damage and reduction of risk of other venous thrombotic events. Our study showed a higher rate of CVT recanalization in patients who received anticoagulation compared with those who did not, however the difference was not statically significant. Due to the small absolute numbers of CVT recurrences and the retrospective design of the study, we could not perform further analyses to correlate with other clinical features.

The most remarkable finding in our study was the high rate of major bleeding complications, in particular ICH. This differs from the lower frequency of ICH complications seen in anticoagulation clinical studies for cancer associated-thrombosis and in other non-cancer population CVT studies [19–22, 13, 18]. We hypothesize that the differences are explained by the provoked (trauma, intracranial tumor, invasive brain procedures, radiation) etiology in our cohort as opposed to anticoagulation only-related bleeding.

Additionally, our data showed that the risk of bleeding was independently increased during the use of warfarin when compared with LMWH. Other investigators have found associations suggesting that overweight population may require higher doses of warfarin to maintain a therapeutic international normalized ratio (INR) [23]. Others have also observed that an elevated body mass index predicts a higher incidence of bleeding during anticoagulation with warfarin [24]. We did not find a statistically differences in bleeding outcomes by the body mass index of the patients. Current guidelines recommend monitoring carefully using the INR in obese and non-obese patients [25–29]. In our study, data on INR and dosage of anticoagulants were not retrieved.

Limitations to our study are the retrospective design; therefore, we could not estimate the effect of anticoagulation monitoring, drug-to-drug interactions, appropriate dosing, and treatment compliance in our outcomes. Additionally, due to the small cohort, our observations are limited, yet suggestive of higher bleeding complications in CVT cancer-population, in particular in those treated with warfarin.

## Conclusions

Our findings are suggestive of a narrow therapeutic index of anticoagulation in cancer-CVT. Careful monitoring of anticoagulation complications are of utmost clinical relevance in cancer patients; in particular those with brain metastasis, invasive procedures, and treatment with warfarin. While our population is exclusively of cancer patients, larger and controlled studies are needed to confirm our observations in that population.
