# Correction to: Thrombosis and bleeding outcomes in the treatment of cerebral venous thrombosis in cancer

**DOI:** 10.1186/s12959-021-00300-y

**Published:** 2021-07-14

**Authors:** Nadia I. Abelhad, Wei Qiao, Naveen Garg, Cristhiam M. Rojas-Hernandez

**Affiliations:** 1 Department of Medicine, University of Texas Health Science Center at Houston, Houston, USA; 2 Department of Biostatistics, University of Texas M.D. Anderson Cancer Center, Houston, USA; 3 Department of Diagnostic Radiology, Division of Diagnostic Imaging, University of Texas M.D. Anderson Cancer Center, Houston, USA; 4 Department of Medicine, Section of Benign Hematology, University of Texas M.D. Anderson Cancer Center, 1515 Holcombe Blvd. Suite, Houston, TX 1464 USA

**Correction to: Thrombosis J 19, 37 (2021)**

**https://doi.org/10.1186/s12959-021-00292-9**

Following publication of the original article [1], it was reported that there was an error in Fig. 1 and in the Abstract.

**Fig. 1:**
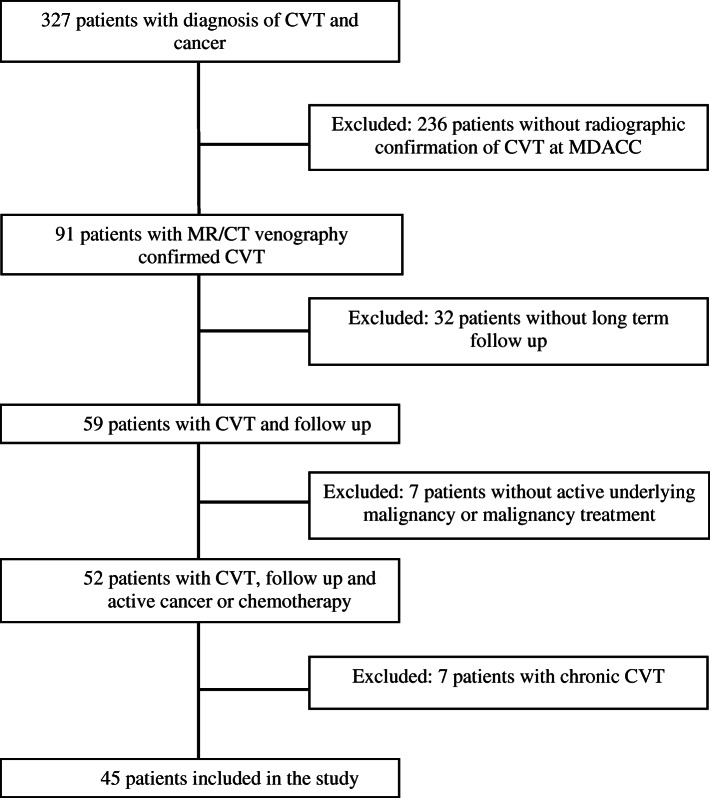
Selection of cancer patients with acute cerebral venous thrombosis (CVT)

In the Abstract it was stated in the Results sub-section that a total of 31 cases were treated with anticoagulation, but the correct total is 33.

In Fig. 1, the original version stated that 13 patients with chronic CVT were excluded, but the correct number is 7. The corrected Fig. 1 is given below.

The original article has been updated.
